# An exploratory study on the presence of *Helicobacter heilmannii* and *Helicobacter billis* in the feces of companion dogs

**DOI:** 10.1002/vms3.765

**Published:** 2022-02-13

**Authors:** Mahdi Fatemi Khader, Mahdi Pourmahdi Borujeni, Naghmeh Moori Bakhtiari, Reza Avizeh

**Affiliations:** ^1^ Department of Food Hygiene, Faculty of Veterinary Medicine Shahid Chamran University of Ahvaz Ahvaz Iran; ^2^ Department of Pathobiology, Faculty of Veterinary Medicine Shahid Chamran University of Ahvaz Ahvaz Iran; ^3^ Department of Clinical Sciences, Faculty of Veterinary Medicine Shahid Chamran University of Ahvaz Ahvaz Iran

**Keywords:** companion dogs, epidemiology, Helicobacter pylori, Helicobacter spp, PCR, zoonotic

## Abstract

**Background:**

Companion animals like dogs play an important role in the lives of many people and are often considered to be members of families, but definitely, any contact with them poses an inherent risk of transmitting zoonotic pathogens. One of these pathogens is the genus *Helicobacter* which is linked to many disorders in human and animal.

**Objectives:**

The aim of this study was to investigate the presence of some zoonotic species of genus Helicobacter in companion dogs.

**Results:**

Through culturing in a special medium, nine samples (9%) were detected as infected (two pure and seven mixed culture). Based on multiplex‐PCR, 13 samples (13%) were infected by *Helicobacter* spp. although none of them were infected by *H. pylori*. Species‐specific PCR indicated that 38.5% or 5/13 of the samples were infected with *H. heilmannii*, while 15.45% or 2/13 of the samples were infected by *H. billis*. Multivariate logistic regression analysis showed that the age factor had a significant effect on *Helicobacter* spp. infection (odds ratio [OR] = 2.42, *p *= 0.01).

**Conclusion:**

This study revealed the negligible faecal transmission of *H. pylori*. Moreover, due to the detection of *H. Heilmannii* and *H. billis* in feces and their association with human gastric diseases, dog owners should be educated about the risks and transmission modes of zoonotic bacterial infections of dogs.

## Graphical abstract

The aim of this study was to determine the prevalence of *Helicobacter* spp. in canine feces by culture and molecular methods. Another objective was to confirm the excretion of *H. pylori*, *H. Heilmannii* and *H. billis* from feces in companion dogs with and without gastrointestinal disorders. This study revealed the negligible faecal transmission of *H. pylori*. Moreover, due to the detection of *H. Heilmannii* and *H. billis* in feces and their association with human gastric diseases, dog owners should be educated about the risks and transmission modes of zoonotic bacterial infections of dogs. The findings of the present study may be useful to clarify the epidemiology of *Helicobacter* spp. in dogs and humans.

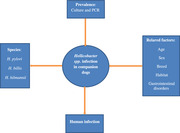

## 1 INTRODUCTION

*Helicobacter pylori*, a Gram‐negative and spiral‐shaped bacterium, was discovered in 1982 (Marshall & Warren, 1984). More than half of the population worldwide may have been infected by this bacterium. However, only 5%–10% of them show clinical symptoms (Goh et al., 2011; Mladenova‐Hristova et al., 2017). Having reviewed 263 published articles on the prevalence of *H. pylori* infection and collecting data from 62 countries, Hooi et al. (2017) showed that Africa has the highest pooled prevalence of *H. pylori* infection (70.1%), whereas Oceania has the lowest prevalence (24.4%). Among the countries, the prevalence of this infection varies from 18.9% in Switzerland to 87.7% in Nigeria. Based on the regional prevalence estimates, there were approximately 4.4 billion individuals with *H. pylori* infection worldwide in 2015 (Hooi et al., 2017). At present, the role of *H. pylori* has been determined in the chronic active gastritis, peptic and duodenal ulcers, gastric adenocarcinoma and gastric mucosa associated lymphoid tissue (MALT) lymphomas. In 1994, the International Agency for Research on Cancer (IARC) categorized *H. pylori* as a class I carcinogen and reported it in 75% of patients with MALT lymphoma and in 60% of those with an increasing risk of gastric cancer (Malfertheiner et al., 2017; Morgner et al., 2000). Also, *H. pylori* infection could be related to some important diseases in humans such as the reduction of the ferritin and iron levels in patients with coronary artery disease and changes in lipid profiles and inflammatory factors, such as pre‐eclampsia as a result of impairing the placental development, and glucose homeostasis in patients with type 2 diabetes (Bonfigl, 2016; Di Simone et al., 2017; Fallah et al., 2016). Furthermore, the infection is directly related to the metabolic syndromes such as high values of triglycerides, body mass index and systolic blood pressure and low HDL (Upala et al., 2016). Following the discovery of *H. pylori*, other spiral bacteria have been observed in many other considered animal species (Dent et al., 1987). In further research employing molecular analysis of the 16rRNA, it was elucidated that these spiral bacteria belonged to the *Helicobacter* genus (Solnick et al., 1993). At present, this genus, with at least 35 species distributed worldwide, can infect human and animals by colonizing in different anatomical regions of the gastrointestinal system such as oral cavity, stomach, intestine and liver. Depending on the place of colonization, it is also divided into gastric and enterohepatic species (Fox & Wang, 2002; Mladenova‐Hristova et al., 2017; Recordati et al., 2009). Gastric *Helicobacter* such as *Helicobacter heilmannii*, *Helicobacter felis*, *Helicobacter salomonis, Helicobacter bizzozeronii*, *Helicobacter cynogastricus* and *Helicobacter pylori* and enterohepatic *Helicobacter* such as *Helicobacter canis*, *Helicobacter billis*, *Helicobacter cinaedi* and *Helicobacter rappini* were isolated from dogs. These species, which are associated with hepatobiliary and gastrointestinal diseases, are transmitted either directly through oral–oral and anal–oral contact or indirectly through water and food in dogs (Buczolits et al., 2003; Ekman et al., 2013; Haesebrouck et al., 2009; Jalava et al., 1997; Jankowski et al., 2016a; Jankowski et al., 2016a; Kubota‐Aizawa et al., 2017; Mladenova‐Hristova et al., 2017; Recordati et al., 2007; Recordati et al., 2009; Rossi et al., 2008; Van den Bulck et al., 2005). *Helicobacter* spp. infection has been reported in 23%–100% of companion dogs and in 70% and 30% of people in both developing and developed countries, respectively (Agüloğlu et al., 2006; Amorim et al., 2015; Chung et al., 2014; Downsett & Kowolik, 2003; Hong et al., 2015; Hwang et al., 2002; Jankowski et al., 2016a, 2016b; Kubota‐Aizawa et al., 2017; Recordati et al., 2009). Despite the high prevalence of this genus, its transmission mechanism in human–human, animal–human, human–animal and animal–animal paths remains unclear (Ekman et al., 2013; Recordati et al., 2007).

The diagnostic methods include invasive and non‐invasive means; the former with high sensitivity and specificity encompasses gastroscopy and collecting a biopsy sample of the gastric mucosa for rapid urea test, histopathological examination, direct Gram staining, microbiological culture, electron microscopy, fluorescent in situ hybridization and PCR. Furthermore, regardless of the risks of gastroscopy and the associated anaesthesia, it should be noted that many veterinarians and clinics do not have the facilities and ability to perform gastroscopy. Therefore, non‐invasive methods, including serology, culture and PCR on blood, saliva and faecal samples are preferred (Haesebrouck et al., 2009; Hong et al., 2015; Pohl et al., 2019; Shinozaki et al., 2002).

*H. pylori* is transmitted from experimentally infected dogs to the uninfected ones once being isolated from gastric mucosa and saliva specimens in dogs (Ekman et al., 2013; Jankowski et al., 2016a, 2016b; Lee et al., 1992; Radin et al., 1990). Also, in our previous study, the apparent seroprevalence of *H. pylori* in related and unrelated individuals with dogs and cats was 72.1% (95% CI: 64.8%–79.4%) and 48.8% (95% CI: 42%–55.6%), respectively. Moreover, the odds of infection in related rather than unrelated individuals was 2.71 (95% CI: 1.73%–4.26%) (Ashrafmodarres et al., 2017).

Therefore, the aim of this study was to determine the prevalence of *Helicobacter* spp. in canine feces by culture and molecular methods. Another objective was to confirm the excretion of *H. pylori* from feces in companion dogs with and without gastrointestinal disorders. The results of the present study may be useful to clarify the epidemiology of *Helicobacter* spp. in dogs and humans.

## 2 MATERIALS AND METHODS

### 2.1 Sample collection

The sample studied in this exploratory study included 50 companion dogs with and 50 dogs without gastrointestinal disorders (existence of diarrhoea, vomiting, constipation and loss of appetite) that were referred to the only veterinary hospital in Ahvaz city of Khuzestan province. Also, on the basis of the history and clinical examinations, the dogs infected or suspected of having other body systems diseases were excluded from this study. In addition, there was no history of drug therapy such as antibiotics and proton pump inhibitors (e.g. omeprazole, lansoprazole and pantoprazole) in any of the dogs (at least 2 weeks before sampling). The city of Ahvaz with a population of about 1,300,000 and approximately 4000 domestic dogs is the capital of Khuzestan province (Census, 2016). Companion dogs in this city are often kept only at homes and have no close contact with other dogs and cats (Didehban et al., 2020). In line with the research, the purpose of the study was explained to the dog owners, and informed consent was taken. Sterile cotton swabs were used for scraping the rectal mucosa, and then placed in a 1.5 ml sterile microtube. Swabs were quickly sent to the microbiology lab and cultured in the selective medium. To isolate the *Helicobacter* spp., a minimum time interval between sampling and culture is crucial due to the possible negative effects on *Helicobacter* viability. In addition, the age, sex, breed, and habitat (the keeping place) of each dog were recorded.

### 2.2 Bacterial isolation

To prepare the selective medium, sheep blood agar and several additional antibiotics were required. Therefore, blood agar base (Biolab Diagnostics Laboratory Inc., Hungary) was prepared and autoclaved for 20 min at 121°C and then tempered to 50°C. Then, antibiotics (vancomycin 10 mg/L, trimethoprim 50 mg/L, ceftiufor 50 mg/L, amphotericin B 2–5 mg/L and polymixin B 3500 IU/L) were aseptically added with constant stirring. Finally, the medium was poured into the petri plates.

Before the culturing process, 200 μl of sterile phosphate‐buffered saline (PBS) was added to each microtube, and then the swab was inoculated in the first region of the plate and streaked by loop in another region. The cultured medium was incubated in the microaerophilic condition at 37°C for 3–5 days.

Subsequently, the suspected colonies were detected and purified in the blood agar medium under the previous condition. Meanwhile, the microscopic examination was conducted via preparing the smear and Gram staining. Finally, the suspected isolates inoculated with skim milk were stored at −70°C until being retrieved for further analysis.

### 2.3 Molecular detection of isolates

#### 2.3.1 DNA extraction

In order for the genomic study of isolates and swab samples, first the DNA extraction was done using the GeneAll DNA kit (GeneAll Biotechnology Co., South Korea) following the manufacturer's instructions. Then, based on genus‐specific and different species‐specific primers, the polymerase chain reaction (PCR) test was carried out for the detection of sample contamination with *Helicobacter* genus and different species such as *H. pylori*, *H. heilmannii* and *H. billis*. Primer sequences and references of each primer are shown in Table 1. For the detection of *Helicobacter* genus and *pylori* species by multiplex PCR, the thermal cycles were set as follows: 1 min at 94°C for denaturation, 2 min at 55°C for annealing and 2 min at 72°C for extension. Thirty‐five repetitions were considered for this stage with an early denaturation at 94°C for 5 min and a final elongation at 72°C for 10 min (Farshad et al., 2004). Each PCR reaction tube contained 25 μl of the reaction mix (consisting of 12.5 μl of Mastermix [Ampliqon, Denmark] with 2 mM MgCl2, 1 μl [10 pmol/μl] of each primer and 5 μl of chromosomal extracted DNA). In order to decrease the negative effects of inhibitory factors, 400 ng/ml of bovine serum albumin (BSA) was added to each reaction (Kreader, 1996).

**TABLE 1:** Goal gene, sequence and size of used primers

Name (gene)	Primer sequence	Size (bp)	Reference
*Helicobacter* genus (16srRNA)	5‐GTA AAG GCT CAC CAA GGC TAT‐3 5‐CCA CCT ACC TCT CCC ACA CTC‐3	389	Choi et al. (2001)
*H. pylori* (isocitrate dehydrogenase)	5‐ATGGCTTACAACCTAAAATTTTACAAAAGCC‐3 5‐TCA CAT GTT TTC AAT CAT CAC GC‐3	1200	Argyros et al. (2000)
*H. billis* (16srRNA)	5 ‐AGAACTGCATTTGAAACTACTTT‐3 5 ‐GGTATTGCATCTCTTTGTATGT‐3	638	Fox et al. (1995)
*H. heilmannii* (ureB)	5´‐GGG CGA TAA AGT GCG CTT G‐3´ 5´‐CTG GTC AAT GAG AGC AGG‐3´	580	Neiger et al. (1998)

The protocols of Fox et al. (1995) and Neiger et al. (1998) were used for the molecular detection of *H. billis and H. heilmannii*, respectively. The products of PCR were analyzed by electrophoresis in 1.5% agarose gel in TAE (tris‐acetate‐EDTA) buffer, visualized by safe‐staining (SinaGen, Iran), illuminated by a UV transilluminator (Uvitech, Germany), and finally documented by a gel documentation apparatus. The standard strain of *H. pylori* (Pasteur Institute, Iran) and sterile distilled water were used as positive and negative controls.

### 2.4 Statistical analysis

The statistical analysis of the data was performed using SPSS (version 16.0; SPSS Inc., Chicago, USA). The association between age (year), sex (female or male), breed (Terrier, Doberman, Dachshund, German Shepherd, Spitz, Siberian Husky, Pitbull or Rottweiler), gastrointestinal disorders (yes/no), habitat (yard/apartment) and *Helicobacter* spp. was analyzed by both *χ* ^2^ test and bivariate and multivariate logistic regression. Bivariate logistic regression models were fit to the data for each potential risk factor. Risk factors associated with BoHV‐1 (*p* ≤ 0.3) in bivariate regression were further analyzed in a multivariate logistic regression model, using a backward, stepwise algorithm. The goodness of fit of the model was determined using the Hosmer and Lemeshow test. The comparison of two diagnostic methods (culture and PCR) was performed by McNemar test and kappa statistic calculation. Differences were considered statistically significant at *p* ≤ 0.05.

## 3 RESULTS

### 3.1 Prevalence of *Helicobacter* spp

In the total number of 100 samples, there were 56 female and 44 male dogs. The mean score and standard deviation of age were 1.55 and 0.84 years, respectively. The relative frequency of Terrier, Doberman, Dachshund, German Shepherd, Spitz, Siberian Husky, Pitbull and Rottweiler breeds was 25%, 17%, 15%, 13%, 10%, 9%, 8% and 3%, respectively. Fifty‐nine percent of dogs were kept in the apartment and the rest were kept in the yard.

**FIGURE 1:**
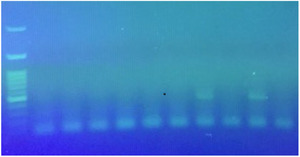
Results of *Helicobacter* spp. and *H. pylori* infection by multiplex‐polymerase chain reaction (PCR). Lane 1: 100 bp ladder, lane 2: negative control, lane 3: *Helicobacter* spp. (389 bp) and *H. pylori* (1200 bp) positive control, lane 6: *Helicobacter* spp. positive samples, lanes 4, 5, 7, 8 and 9: negative samples

**FIGURE 2:**
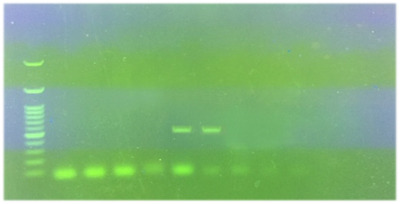
Results of *H. heilmannii* polymerase chain reaction (PCR) detection. Lane 1: 100 bp ladder, lane 2: negative control, lane 3: positive control (580 bp), lanes 5 and 6: positive sample, lanes 4, 7 and 8: negative samples

**FIGURE 3:**
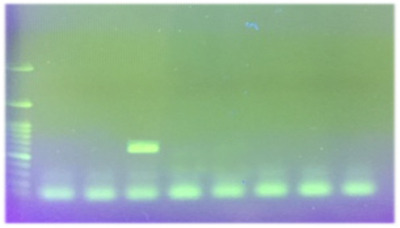
Results of *H. billis* polymerase chain reaction (PCR) detection. Lane 1: 100 bp ladder, lane 2: positive control (638 bp), lane 3: negative control, lane 4: positive sample, lanes 5, 6, 7 and 8: negative samples

According to the culture and biochemical test, nine of the samples (9%) were positive, but two of them were pure and the rest were a maximum mix of two colonies (*Helicobacter* and *campylobacter*). Eighty‐nine percent (eight out of nine positive samples) of samples were collected from dogs with gastrointestinal disorders. The consistency of the results of culture and PCR was demonstrated in nine samples. Four samples shown negative by culture but positive by PCR were isolated from the dogs without gastrointestinal disorders. There was no significant difference between the two diagnostic methods (*p *> 0.05). Besides, kappa statistic was 0.8 (*p *< 0.001). The molecular prevalence rate of *Helicobacter* spp. was 13% (95% CI: 6.4%–19.6%); however, none of them was detected as *H. pylori* by multiplex‐PCR (Figure 1). In *H. heilmannii* species‐specific PCR, 38.5% (5/13 samples), and in *H. billis* species‐specific PCR, 15.4% (2/13 samples) were positive. In all the positive samples, one single *Helicobacter* species was present. Eighty percent of *H. heilmannii* positive cases were related to dogs with gastrointestinal disorders, and 100% of *H. billis* positive cases were related to dogs without gastrointestinal disorders. The results of *H. heilmannii* and *H. billis* PCR are displayed in Figures 2 and 3, respectively.

### 3.2 Risk factors of *Helicobacter* spp. infections

The statistical analysis showed that the infection was correlated with age orders and increased with aging (*p *< 0.05). Univariate logistic regression revealed the odds of infection between the age, based on year, and the disease to be 2.42 (95% CI: 1.22%–4.79%); as one year of age increased, the odds of infection increased by 142%. Moreover, 12.5% of fluctuation in the infection was justified by the age factor (Table 2). The prevalence of *Helicobacter* spp. in male and female dogs was found to be 15.9% and 10.7%, respectively; however, the *χ* ^2^ test showed that the difference was not significant (*p *> 0.05). The odds of infection in males was 1.58 (95% CI: 0.49%–5.08%) compared to those of females. Furthermore, 1.1% of fluctuation in infection was justified by the gender factor (Table 2). The relative frequency of positive cases varied from 0% to 33.3% in the studied breeds, but this difference is not statistically significant (*p *> 0.05). However, 26.2% of fluctuation of infection was justified by the breed (Table 2). The *χ* ^2^ test showed that there was no statistical relationship between the infection and the habitat (*p *> 0.05). The odds of infection in dogs kept in apartments was 1.13 (95% CI: 0.34%–3.74%) compared to the dogs kept in the yard. Besides, 0.1% of fluctuation in infection was justified by habitat (Table 2). The statistical analysis showed that there was no statistical relationship between the infection and gastrointestinal disorders (*p *> 0.05). The odds of infection in dogs with gastrointestinal disorders was 1.71 (95% CI: 0.22%–5.66%) compared to dogs without gastrointestinal disorders. That is, 1.5% of fluctuation in infection was justified by gastrointestinal disorders (Table 2).

**TABLE 2:** Prevalence of *Helicobacter* spp. in dogs in the southwest of Iran based on age, sex, breed, keeping place and gastrointestinal disorders

Category	Groups	Prevalence	OR	95% CI for OR	*p*‐Value
Age (year)	<2**^b^**	5.3% (3/57)			
	≥2^a^	23.3% (10/43)	2.42	1.22–4.79	<0.05
Sex	Female^a^	10.7% (6/56)	1	–	–
	Male^a^	15.9% (7/44)	1.58	0.49–5.08	>0.05
Breed	Doberman^a^	0% (0/17)	–	–	–
	Spitz^a^	0% (0/10)	–	–	–
	Pitbull^a^	0% (0/8)	–	–	–
	German Shepherd ^a^	7.7% (1/13)	1	–	–
	Siberian Husky ^a^	11.1% (1/9)	1.5	0.08–27.61	>0.05
	Terrier ^a^	24% (6/25)	3.79	0.41–35.49	>0.05
	Dachshund ^a^	26.7% (4/15)	4.36	0.42–45.26	>0.05
	Rottweiler ^a^	33.3% (1/3)	6	0.26–140.05	>0.05
Habitat	Yard^a^	12.2% (5/41)	1	–	–
	Apartment^a^	13.6% (8/59)	1.13	0.34–3.74	>0.05
Gastrointestinal disorders	No^a^	10% (5/50)	1	–	–
	Yes^a^	16% (8/50)	1.71	0.52–5.66	>0.05

*Note*: The different lowercase letters in each variable represent a significant difference.

Abbreviations: CI, confidence interval; OR, odds ratio.

Multivariate logistic regression showed that 43.7% of the fluctuation of infection was justified by the factors such as age, sex, breed, gastrointestinal disorders and habitat. However, in backward stepwise logistic regression, only the age factor was significantly related to the infection (*p* = 0.012) (Hosmer and Lemeshow test: *χ* ^2^ = 4.11, *df* = 4, *p *= 0.39).

## 4 DISCUSSION

This pioneering epidemiological survey, in addition to determining *Helicobacter* spp. in feces, identified some of the factors associated with its occurrence in companion dogs in the southwest of Iran. Understanding the epidemiology of this organism is definitely an essential key to establishing appropriate prevention strategies.

In this study, using culture and multiplex‐PCR methods to assess faecal samples, *Helicobacter* spp. was identified in 9% and 13% of the dogs, respectively; however, this observed difference was not statistically significant. According to other studies, prevalence of *Helicobacter* spp. DNA in feces was reported to be 100% by Ekman et al. (2013), 62.5% by Hong et al. (2015) and 23.3% by Jankowski et al. (2016a). In gastrointestinal biopsies of dogs with gastritis by PCR, the prevalence of *Helicobacter* spp. in Japan and Poland was reported as 34.7% and 100%, respectively (Jankowski et al., 2016a; Kubota‐Aizawa et al., 2017). The quantitative polymerase chain reaction (qPCR), histological, histochemical and immunohistochemical evaluations on gastric samples revealed that *Helicobacter* spp. was present in 47.8%, 65.2%, 75.4% and 82.6% of dogs, respectively (Amorim et al., 2015). The relative frequency of *Helicobacter* spp. in oral cavity (dental plaque and saliva) samples was 71.1%–100% (Ekman et al., 2013; Jankowski et al., 2016a; Recordati et al., 2007a). In Brazil, *Helicobacter* spp. infection has been reported as 94.7% and 100% by rapid urease test and histological analysis (Okubo et al., 2017). The large discrepancy in the frequency percentage of *Helicobacter* spp. may depend on the diagnostic method, specimen type, sample size and management and environmental factors (Chung et al., 2014; Falsafi et al., 2009; Hong et al., 2015; Kabir, 2001; Shinozaki et al., 2002; Smith et al., 2012). The detection of *Helicobacter* spp. in faecal samples is performed by microbiological culture, determination of anti‐*Helicobacter* antibodies and bacterial DNA using PCR (Falsafi et al., 2009; Mishra et al., 2008; Smith et al., 2012). Microbiological cultures are not performed routinely to diagnose *Helicobacter* spp. in faecal samples since the sensitivity of this method is low, and *H. pylori* can be difficult to culture. Furthermore, faecal samples have a high concentration of other microorganisms and a low concentration of gastric *Helicobacter* spp.; in addition to this, the long‐time passage of bacteria through the gastrointestinal tract has harmful effects on its viability (Falsafi et al., 2009; Smith et al., 2012). The PCR can be used to detect *Helicobacter* spp. in faecal samples. The sensitivity and specificity of this method are between 69% and 94% and 97.1% and 100%, respectively. However, factors such as the small amount of gastric *Helicobacter* spp. in feces, a degradation of bacterial DNA in the large intestine, the presence of polymerase inhibitors such as complex polysaccharides, the DNA extraction method and type of PCR used affect its validity. Moreover, the PCR does not require a high concentration of bacteria and/or the presence of live bacteria. Furthermore, the use of a semi‐nested or nested‐PCR increases the accuracy of this method compared to the classical PCR (Mishra et al., 2008; Smith et al., 2012; Tonkic et al., 2012). According to Prachasilpchai et al. (2007), the percentage frequency of *Helicobacter* spp. detected by haematoxylin and eosin stain (H&E), Warthin Starry stain (WSS), immunohistochemistry (IHC) and PCR in canine stomach turned out to stand at 17.3%, 46.7%, 30.7% and 10.7%, respectively. Although the detection of *Helicobacter* spp. revealed a statistically meaningful difference between H&E and WSS, IHC and H&E and PCR and H&E, the result of PCR was not in stark contrast with that of WSS and IHC. Despite the fact that IHC was proved to be much more sensitive in pinpointing infection compared to H&E and WSS, it is relatively costly and demanding in terms of time and experience; thus, it is not being deployed for routine tests. However, H&E staining may have low sensitivity when few bacteria are present, so the use of special stains such as WSS facilitates histological identification of bacteria (Dunn et al., 1997). The recognition of the particular species or strains of *Helicobacter* is feasible via PCR due to being a straightforward, precise, time‐saving, automatic and highly efficient method. PCR is applicable to a wide array of samples ranging from gastric biopsies, gastric juice and dental plaque to feces. The sensitivity of this method is considerably higher than that of histology, bacterial culture and urease evaluation although the type of primer used can alter its sensitivity (Prachasilpchai et al., 2007; Sabbagh et al., 2019; Simpson et al., 1999). Finally, not a single ‘gold‐standard’ method exists for the detection of the infection induced by *Helicobacter* spp.; thus, its confirmation requires the joint application of at least two tests (Jankowski et al., 2017; Patel et al., 2014). Ekman et al. (2013) and Hong et al. (2015) used laboratory dogs that were kept together in the same kennel, thus facilitating the transmission of the *Helicobacter* spp. compared to the dogs living alone. In addition, the large percentage of *Helicobacter* spp. may be due to the small sample size. For example, the sample size in the surveys of Ekman et al. (2013), Hong et al. (2015) and Jankowski et al. (2016a) was 14, 8 and 30 dogs, respectively, which is much smaller than the sample size in the present study (100 dogs). Specimen type is also influential. For example, Jankowski et al. (2016a) showed that the frequency of *Helicobacter* spp. in saliva and gastric biopsy specimens was 76.6% and 100%, respectively. Also, Recordati et al. (2007) detected *Helicobacter* spp. DNA by nested PCR in 94.7% of gastric biopsies, 44.7% of dental plaque and 50% of saliva samples in dogs. The high prevalence shown in the studies by Hong et al. (2015) and Jankowski et al. (2016a) may be related to the fact that all dogs under the experiment had gastritis, whereas in the present study, only 50% of dogs had gastrointestinal disorders. In addition, the low prevalence of Helicobacter spp. in this study may reflect the regional differences such as management and environmental factors between Iran and other countries because the dogs in this study, with no previous antibiotic therapy background, were kept only at homes and did not have any close contact with other dogs and cats.

In this study, the relative frequency of *H. heilmannii*, *H. billis* and *H. pylori* was detected to be 38.5%, 15.4% and 0%, respectively. *H. billis* is grouped in enterohepatic *Helicobacter*; thus, its isolation was expected from feces but *H. pylori* and *H. heilmannii* are grouped in gastric *helicobacter*, so their isolation may not be expected. Also, in all the positive dogs, one single *Helicobacter* species was present. Similarly, the frequency percentage of *H. canis* and *H. billis* in faecal samples of 14 clinically normal Beagles held for educational purposes was 100% and 14.3%, respectively, but that of *H. py*lori, *H. felis*, *H. salomonis* and *H. bizzozeronii* was 0% (Ekman et al., 2013). Moreover, the prevalence of *H. heilmannii* and *H. salomonis* in faecal samples of 30 dogs with gastritis was 71.4% and 28.6%, respectively, but that of *H. felis*, *H. pylori* and *H. bizzozeronii* was 0% (Jankowski et al., 2016a). Hong et al. (2015) reported that the frequency of *H. heilmannii* and *H. felis* was 37.5% and 25%, respectively. The prevalence of *H. heilmannii*, *H. salomonis*, *H. bizzozeronii*, *H. pylori* and *H. felis* in saliva of 30 dogs with gastrointestinal disorders has been reported to be 95.7%, 17.4%, 13%, 8.7% and 4.4%, respectively (Jankowski et al., 2016a). *H. pylori* infection in dogs is rare. For example, in one study, *H. pylori* infection was found in 144 dogs with gastrointestinal diseases (Kubota‐Aizawa et al., 2017). In addition, Abdel‐Raouf et al. (2014) reported 41.1%, 42.9% and 50% infection caused by *H. pylori* in stool, saliva and stomach juice samples of dogs by microbiological culture, respectively; in the same vein, Elhariri et al. (2017) reported 37.2% infection caused by this bacterium in serology. However, the failure of the serological methods in differentiating between the current infection and the previous exposure is regarded as the main shortcoming due to resulting in misinterpretation (Sabbagh et al., 2019). In addition, another source of misinterpretation is the cross‐reacting antigens, especially flagellar proteins, residing between *H. pylori* and campylobacters (Mégraud & Lehours, 2007).

In the present study, age unlike gender, breed, gastrointestinal disorders and habitat was significantly related to the infection caused by *Helicobacter* spp. Regarding the age, the higher infection rate in older dogs was probably due to a greater exposure to the *Helicobacter* spp. over time. In the present study, the relative frequency of positive samples in dogs with gastrointestinal disorders was not statistically higher than those without gastrointestinal disorders, thus proving that *Helicobacter* infection could be asymptomatic in dogs. In several studies, no significant relationship was detected between infection and age, gender, gastrointestinal disorders and domestic habitat (Ekman et al., 2013; Elhariri et al., 2017; Okubo et al., 2017; Recordati et al., 2007). However, Kubota‐Aizawa et al. (2017) showed that dogs positive for *Helicobacter* spp. had a significantly higher frequency of chronic diarrhoea than the negative dogs. A clear relationship between the presence of *Helicobacter* spp. and both mild to moderate epithelial injury and mild to moderate intraepithelial lymphocyte infiltration of the canine stomach has been reported by Amorim et al. (2015). Hwang et al. (2002) showed that the total positive rate in clinically abnormal dogs is significantly higher compared to the clinically normal dogs in the urease test and PCR. It should be kept in mind that although the veterinary hospital in Ahvaz is the most important and the largest veterinary centre in southwest of Iran, the findings might not reflect the whole dog population in Ahvaz. Also, due to the significant size of the sample in this study (100 dogs and bacterial culture as well as direct PCR on their feces) and limited financial resources and time, only the prevalence of three zoonotic species of *Helicobacter* spp. was evaluated, and thus other species such as *H. bizzozeronii, H. salomonis, H. canis* and *H. felis* will be investigated in the upcoming research.

## 5 CONCLUSION

The rate of *Helicobacter* spp. infection in feces of companion dogs with and without gastrointestinal disorders is scant but noticeable, especially in zoonotic species such as *H. billis* and *H. heilmannii*. Canine feces can be a potential source of *Helicobacter* spp.; therefore, dog owners should be educated about the risks and transmission modes of bacteria by veterinarians in the southwest of Iran. In addition, the present study indicated that the transmission risk of *H. pylori* through feces of dogs appears to be negligible.

## CONFLICT OF INTEREST

The authors declare no conflict of interest.

## ETHICS STATEMENT

This study was ‘observational study’ and the research protocol was reviewed and approved by research committee of the Faculty of Veterinary Medicine, Shahid Chamran University of Ahvaz and documented by the number 915824. Before beginning work on the present study, the dog owners were briefed on the purpose of the research and written informed consent was obtained from the owners for the participation of their animals in this study. During collecting rectal swabs, these animals were not disturbed.

## AUTHOR CONTRIBUTIONS

*Data curation (equal), investigation (equal), methodology (equal), validation (equal), visualization (equal) and writing—review and editing (equal)*: Mahdi Fatemi Khader. *Conceptualization (equal), data curation (equal), formal analysis (lead), investigation (equal), methodology (equal), project administration (lead), resources (equal), software (equal), supervision (lead), validation (equal), visualization (equal), writing—original draft preparation (equal) and writing—review and editing (equal)*: Mahdi Pourmahdi Borujeni. *Conceptualization (equal), data curation (equal), investigation (equal), methodology (equal), resources (equal), software (equal), validation (equal), visualization (equal), writing—original draft preparation (equal) and writing—review and editing (equal)*: Naghmeh Moori Bakhtiari. *Data curation (equal), investigation (equal), methodology (equal), resources (equal) and writing—review and editing (equal)*: Reza Avizeh.

### PEER REVIEW

The peer review history for this article is available at https://publons.com/publon/10.1002/vms3.765.

## Data Availability

The data that support the findings of this study are available from the corresponding author upon reasonable request.
